# Cellular dynamics underlying regeneration of appropriate segment number during axolotl tail regeneration

**DOI:** 10.1186/s12861-015-0098-1

**Published:** 2015-12-09

**Authors:** Carr D. Vincent, Fabian Rost, Wouter Masselink, Lutz Brusch, Elly M. Tanaka

**Affiliations:** Max-Planck Institute of Molecular Cell Biology and Genetics, Pfotenhauerstraße 105, 01307 Dresden, Germany; Technische Universität Dresden, DFG Center for Regenerative Therapies, Fetscherstraße 105, 01307 Dresden, Germany; Technische Universität Dresden, Center for Information Services and High Performance Computing, and Center for Advancing Electronics Dresden (cfaed), 01062 Dresden, Germany

**Keywords:** Salamander, Tail, Regeneration, Size control, Cell cycle, Myotome, Segmentation

## Abstract

**Background:**

Salamanders regenerate their tails after amputation anywhere along their length. How the system faithfully reconstitutes the original number of segments and length is not yet known.

**Methods:**

To gain quantitative insight into how the system regenerates the appropriate length, we amputated tails at 4 or 16 myotomes post-cloaca and measured blastema size, cell cycle kinetics via cumulative Bromodeoxyuridine (BrdU) incorporation and the method of Nowakowski, and myotome differentiation rate.

**Results:**

In early stages until day 15, blastema cells were all proliferative and divided at the same rate at both amputation levels. A larger blastema was formed in 4th versus 16th myotome amputations indicating a larger founding population. Myotome differentiation started at the same timepoint in the 4th and 16 th level blastemas. The rate of myotome formation was more rapid in 4th myotome blastemas so that by day 21 the residual blastema from the two amputation levels achieved equivalent size. At that time point, only a fraction of blastema cells remain in cycle, with the 4th myotome blastema harboring double the number of cycling cells as the 16th myotome blastema allowing it to grow faster and further reconstitute the larger number of missing myotomes.

**Conclusions:**

These data suggest that there are two separable phases of blastema growth. The first is level-independent, with cells displaying unrestrained proliferation. In the second phase, the level-specific growth is revealed, where differing fractions of cells remain in the cell cycle over time.

## Background

How animals regulate organ size and proportion is an enduring question in biology. Regeneration is a particularly interesting context to ask this question, as the organ must regenerate anew in the context of an already developed organism. Furthermore, regeneration of the vertebrate limb or tail, for example, show appropriate level-specific growth control and morphogenesis to replace only the missing structure. How the growth control is achieved and linked to differentiation of tissues is not yet understood.

Studies in the salamander limb have carefully measured proliferation rates and distributions to investigate patterns of growth control. The most recent studies concur that there is a uniform distribution of mitotically active cells along the proximal-distal axis of the limb blastema and that there is no difference in cell cycle rate between upper arm and wrist blastemas. The studies, however, have come to divergent conclusions about the timing of differentiation, with one study concluding that upper arm and lower arm limb regenerates progressed through the different stages at the same time, whereas other studies concluded that the upper arm blastemas were delayed in differentiation by 1–2 days [1, 2]. When mitotic indices were measured in *Notophthalmus viridescens* and *Ambystoma maculatum*, there was a noticeable decline during the time course of limb regeneration suggesting either a slow down or withdrawal from the cell cycle during regeneration [2, 3]. However, these parameters were similar between upper arm and lower arm blastemas [2]. Therefore, how the position of the cut influences growth kinetics on a cellular scale had not been resolved. Analysis of differentiation kinetics in the limb is, however, complicated since the morphology of the upper arm skeleton and muscle differs from the lower arm and hand, which may lead to intrinsic differences in differentiation kinetic unrelated to a generic proximal/distal position.

Tail regeneration in salamanders represents an excellent system to study growth control since the tail regenerates the correct number of tail segments following cuts along the length of the tail and the segments are uniform in composition and morphology [4, 5]. In previous studies, the change in regenerate length (blastema + newly differentiated tissue) in tails cut at two different amputation planes was measured. The authors concluded that the two types of tails progressed through the different regeneration stages at the same time, indicating that the timing of major events may not be dependent on the amount of tissue that must be regenerated. In these studies, the authors did not follow the size of the blastema versus the size of newly differentiating tissue so the kinetics of segment differentiation with respect to maintenance (and the relative size) of an undifferentiated blastema cell zone in the two types of regenerate remain unknown. Furthermore, cell cycle rates and total cell numbers in the regenerating tissue were not measured. Finally, none of the previous studies of limb or tail regeneration estimated the size of the progenitor pool that is recruited into the blastema, and whether the spatial zone from which the source cells are recruited varies with the amputation level. This lack of quantitative cell data has limited the modelling of this growth control phenomenon.

Here we acquired quantitative cellular growth and differentation measurements of tails regenerating from two different amputation planes, to provide a framework for modeling growth control during tail regeneration.

We dynamically tracked the course of myotome differentiation versus blastema size in terms of cell number in animal cohorts cut at proximal versus distal amputation planes. This allowed us to determine the important points of growth regulation that result in regeneration of a tail of the correct size. Furthermore we provide cell cycle length measurements and total cell number at critical timepoints of regeneration. We find that growth control is biphasic. In the early phase, there is no position dependent growth. All blastema cells in the early blastema are dividing at a rapid rate. Later, however, after the initial onset of myotome formation, blastemas show level-specific differences with a larger proportion of cells remaining in the cell cycle in the blastema that must still regenerate more tail tissue. Using a mathematical modeling approach, we infer the size of the activated progenitor population and find its spatial extent proximal to the amputation plane to be position-independent and matching well with that reported for the spinal cord, suggesting universal activation signals. This data constrains models that can be built to describe size control during tail regeneration and provides a strong framework for molecular studies on this topic.

## Results

**Fig. 1:**
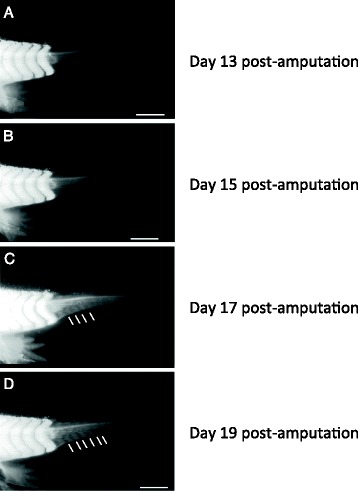
Myotome organization in the regenerating tail of the axolotl. **a**–**d**. Photomicrographs of regenerating CAGGS:GFP transgenic axolotl tails. Myotomes are visible because CAGGS:GFP is strongly expressed in muscle tissue. Regenerating tails 13, 15, 17 and 19 days post-amputation at 4th myotome posterior to the cloaca (proximal cut). **a**. Lack of organized muscle tissue at day 13. **b**. Onset of myotome formation at day 15. **c**. Clearly countable myotomes at day 17. White lines demarcate some of the intermyotome regions. **d**. More myotomes formed by day 19. Scale bars, 2,5 mm

**Fig. 2:**
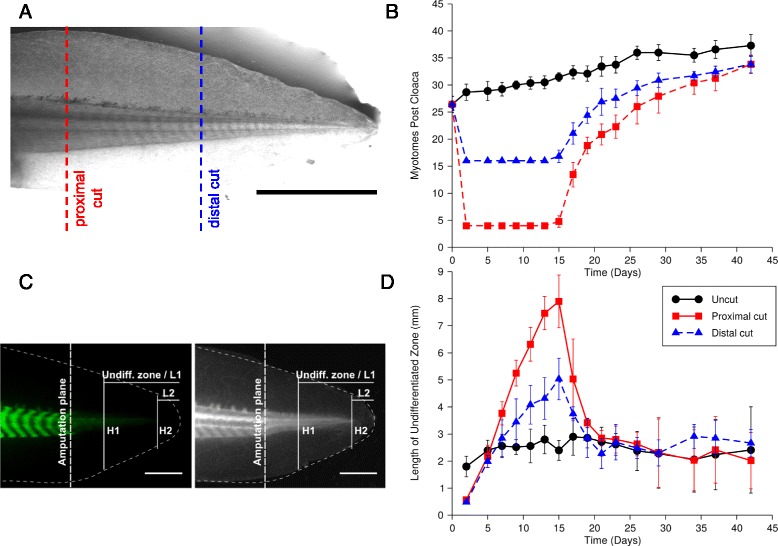
Myotome organization in proximal and distal regenerates starts at a fixed time after amputation, but blastema size differs depending on amputation level. **a**. Tails were amputated proximally (at the 4th myotome posterior to the cloaca, red) or distally (16th myotome posterior to the cloaca, blue). Scale bar, 5 mm. **b**. Regenerating tails were monitored over time and the number of visible myotomes was scored. **c**. Fluorescent and brightfield images of regenerating tail depicting the parameters measured in Fig. 2d and Fig. 3. Scale bar, 1 mm. **d**. The length of regenerated tissue posterior to the last myotome (called blastema) was measured until the tail tip (denoted L1 in Fig. 2c) as a function of time after amputation. Values shown at each time point are the mean of at least six animals; error bars represent standard deviations from the mean. Results shown are representative of two independent experiments

To dynamically monitor the process of tail regeneration including blastema formation and myotome differentiation, we made measurements in cohorts of live, 3 cm long axolotl “white” mutant larvae, expressing the CAGGS:GFP transgene, where the myotome organization is visible under the light microscope (Fig. 1). We first sought to quantitatively compare the process of myotome re-formation in regenerating tails to the process of myotome organization during normal tail growth, since these larvae are still growing in overall body size. In normally developing tails, we observed that myotome number increased at a uniform rate of approximately 1 new myotome every 4 days during the course of the experiment (Fig. 2b, uncut shown as black curve). In contrast, the rate of myotome organization in the regenerating tail was not uniform. We observed that after amputation at the 4th myotome caudal to the cloaca, no new myotomes were observed in the regenerating tissue until after 15 days. During this time there was significant outgrowth of blastema tissue. However, after 15 days, we observed a sudden increase in myotome organization in the regenerated tissue (Fig. 1c). We noted that the precision and regularity of the myotomes varied from animal to animal and could be visually scored as segments, though they were not as perfectly formed as the regular structure of tail myotomes observed in development.

### Onset of myotome organization occurs at the same time in proximal versus distal regenerates

We used this assay to measure the kinetics of myotome organization in tails cut at the 4th (proximal) or 16th (distal) myotome post-cloaca in cohorts of animals (Fig. 2a). Interestingly, we observed that onset of myotome organization occurred at the same time after amputation at proximal and distal levels (Fig. 2b). This onset of myotome organization began at day 15 post-amputation, and proceeded at a rapid rate in both proximal and distal blastemas until the regenerated tails approached the number of myotomes and corresponding formation rate present in unamputated tails. The approach to homeostatic rates occurred gradually, with maximum rate from day 15 to approximately day 19 (3.5 ± 0.2 new myotomes per day for the proximal and 1.9 ± 0.2 for the distal regenerate, mean ± SEM over linear regression results of ≥6 individual animals per condition, t-test between proximal and distal: p < 10^−4^ showed significant dependence on cut position).

### Differences in blastema properties in proximal versus distal amputations

**Fig. 3:**
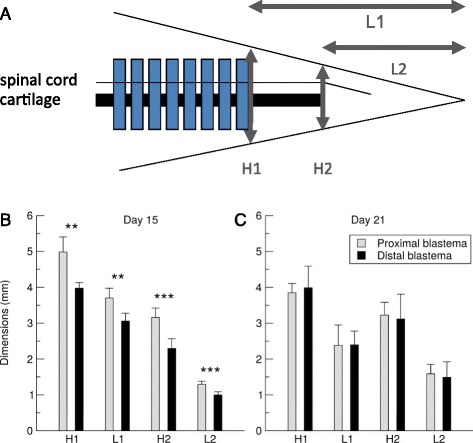
Blastema dimensions and amputation level are dependent on day 15 and independent by day 21. **a**. Sketch defining heights H1, H2 and lengths L1, L2 of blastemas measured from the last visible myotome to the tail tip, and from the tip of the cartilage rod to the tail tip, respectively. **b**–**c**. Values shown are the mean measurements of at least six animals (two independent experiments from that shown in Fig. 2); error bars represent standard deviations from the mean. **b**. On day 15, all blastema dimensions are significantly different between proximal and distal amputations (results of t-tests given above each data pair). **c**. There are no significant differences between the dimensions of blastemas from proximal and distal amputations at day 21

Given that the proximal blastema ultimately gives rise to a larger number of myotomes, we then examined the blastema for properties that might account for this difference in myotome formation capacity. To determine whether the proximal versus distal blastemas have similar or different dimensions at myotome onset, we measured the length of the undifferentiated blastema over time (Fig. 2c,d). The undifferentiated blastema was defined as the length of regenerating tissue posterior to the last myotome until the tail tip (denoted L1 in Fig. 3a, see Fig. 2c). At day 15, the proximal blastemas were significantly longer in anterior/posterior dimension compared to distal blastemas (one way ANOVA test: p < 10^−7^ and post-hoc Tukey’s HSD tests: p < 0.001 showed that all three conditions including uncut were pairwise significantly different). 4th myotome blastemas had an average length of 7.9 ± 1.0 mm while 16th myotome blastemas were 5.0 ± 0.8 mm. Interestingly, by day 21, after the initial burst of myotome formation, the length of undifferentiated blastema was similar between proximal and distal samples (2.9 ± 0.3 mm and 2.3 ± 0.5 mm, respectively) (Fig. 2d) consistent with our measurements that myotomes formed faster in the proximal versus distal cut (Fig. 2b). Moreover, at day 21, the length of undifferentiated blastema in regenerating tails was similar to the distance from the last myotome to the tip of the tail in unamputated, growing tails (average length of 2.7 ± 0.5 mm, one way ANOVA test of the three conditions: p = 0.13 showed no significant differences). We compared the height and length of blastemas in two independent experiments with separate animals (Fig. 3). At day 15, not only the length but also blastema height differed significantly between 4th versus 16th myotome blastemas (Fig. 3b) whereas no significant difference in blastema dimensions was found at day 21 (Fig. 3c).

Although by day 21, proximal versus distal blastemas had similar dimensions, we observed that myotomes in the proximal samples were still forming at a significantly faster rate (0.58 ± 0.04 myotomes/day, mean ± SEM over linear regression results of ≥6 individual animals for days 21–42) than the distal samples (0.33 ± 0.05 myotomes/day, t-test between proximal and distal: p < 0.005), suggesting a higher growth rate of proximal versus distal blastemas (Fig. 2b). This difference in growth rate suggests two possibilities: (1) cells in the proximal blastemas are progressing through the cell cycle at a faster rate than cells in distal blastemas, or (2) a larger proportion of cells in proximal blastemas are cycling at day 21. We further wanted to know if such cell cycle differences accounted for the formation of a larger proximal blastema at day 15.

### Cell cycle parameters are similar in the early blastemas but differ in the late blastemas originating from proximal versus distal cuts

To measure the cell cycle parameters of blastema cells, we implemented the cumulative BrdU labeling method described by Nowakowski et al. where a series of samples are cumulatively labeled with Bromodeoxyuridine (BrdU) for differing lengths until saturation of label is achieved [6]. From such data the total growth fraction, the total cell cycle length, and the length of S-phase in the cell population can be determined. Although the simple deduction of these three cell cycle parameters relies on additional assumptions (see Methods), we employed it as a robust framework to probe this complex system for differences in cell cycle properties between proximal versus distal blastema cells at two time points (described further below). We further limited our analysis to the mesenchymal blastema cells, excluding the cells that comprise the epidermis, spinal cord, and cartilage of the regenerating tail. We expect the growth characteristics of the blastema to derive primarily from the positional behavior of the total mesenchymal population. Although it is likely that myotomes form from a specific subset of these mesenchymal cells, we were unable to combine a molecular marker for myogenic precursors with the BrdU labelling protocol for analysis. In our hands, EdU labelling resulted in profound alterations in the cell cycle behavior of our cells, and was therefore not used in these studies.

**Fig. 4:**
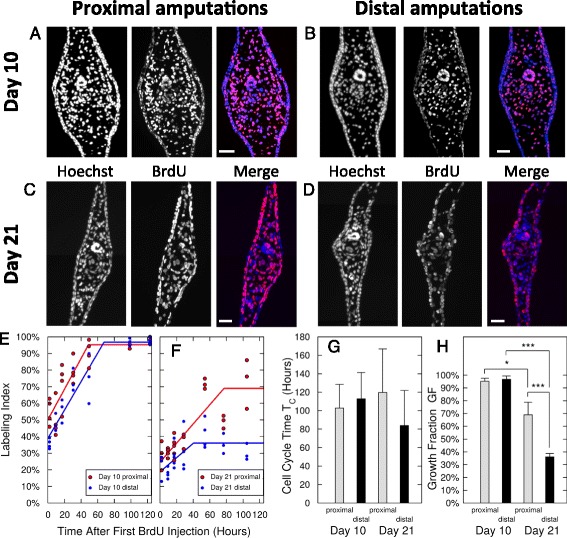
Cell cycle analyses of day 10 and day 21 tail blastemas after proximal and distal amputations reveal specific growth fractions and uniform cell cycle lengths. **a**–**d**. Representative photomicrographs of 10 day-proximal (**a**), 10 day-distal (**b**), 21 day-proximal (**c**) and 21 day-distal (**d**) blastemas, each cumulatively labeled for 105 h. **a**–**b**. Essentially all cells in both mesenchymal blastemas are BrdU+. **c**–**d**. The majority of nuclei in the 21 day-proximal-mesenchymal blastema (**c**.) but only a minority in the 21 day-distal-mesenchymal blastema (**d**.) are still positive for BrdU. **e**–**f**. BrdU labeling index (dimensionless fraction of BrdU+ cells) as time courses of cumulative BrdU treatment starting at day 10 (**e**.) and day 21 (**f**.). Each data point represents an individual animal (three to five per time point). Solid curves show best-fitting proliferation models (see Methods). **g**–**h**. Cell cycle parameters derived from model fits to the time courses of the BrdU labeling index. **g**. The cell cycle lengths *T*_*C*_ (all parameters reported as mean ± 68 % confidence interval) of all four conditions are very similar with *T*_*C*_ = (103 ± 26) hours for 10 day-proximal, *T*_*C*_ = (112 ± 29) hours for 10 day-distal, *T*_*C*_ = (119 ± 47) hours for 21 day-proximal and *T*_*C*_ = (84 ± 38) hours for 21 day-distal blastemas, none of the differences is statistically significant. The S-phase duration *T*_*S*_ also was very similar for all four conditions with *T*_*S*_ = (54 ± 17) hours for 10 day-proximal, *T*_*S*_ = (45 ± 14) hours for 10 day-distal, *T*_*S*_ = (42 ± 17) hours for 21 day-proximal and *T*_*S*_ = (43 ± 28) hours for 21 day-distal blastemas. **h**. The fraction of proliferating mesenchymal cells (GF) decreased significantly from day 10 to 21 (p < 0.001 for distal and p < 0.05 for proximal blastemas) and at day 21 was significantly lower for distal than for proximal blastemas (p < 0.001) with GF = (95 ± 2) % for 10 day-proximal, GF = (97 ± 3) % for 10 day-distal, GF = (69 ± 10) % for 21 day-proximal and GF = (36 ± 3) % for 21 day-distal blastemas. Scale bars in (**a**–**d**), 100 μm

To compare the growth properties of proximal versus distal blastema cells, we performed the cumulative BrdU measurements at two different time points. One set of measurements was made starting at 10 days post-amputation, as the blastema is arriving at maximal length, prior to myotome organization (Fig. 4a,b,e). The second set of measurements was made at day 21 when proximal and distal blastema dimensions are similar but myotome formation is still faster in the proximal regenerate (Fig. 4c,d,f). In the 10 day blastema, based on long-term BrdU incorporation, we found that the total number of cycling cells (GF) in the mesenchyme of both proximal and distal blastemas was indistinguishable (GF = 95 ± 2 % proximal, GF = 97 ± 3 % distal, all parameters reported as mean ± 68 % confidence interval), indicating that nearly all of the mesenchymal cells in proximal and distal blastemas are actively cycling at day 10 to 15. Based on the time course of cumulative BrdU incorporation, we calculated the average cell cycle length for the mesenchymal cells in blastemas to be 103 ± 26 h for proximal blastemas, and 112 ± 29 h for distal blastemas. These data indicate that at early stages, proximal and distal blastema cells have similar cell cycle characteristics (Fig. 4g,h).

In contrast, our measurements made from day 21 samples showed a significant difference in growth characteristics (p < 0.001 for proximal vs. distal GF). The growth fraction was 69 ± 10 % in proximal blastemas and 36 ± 3 % in distal blastemas (Fig. 4c,d,h). While these GFs were significantly lower than their day 10 counter parts, the total cell cycle length was, however very similar between proximal and distal blastema samples (119 ± 47 versus 84 ± 38 h, respectively) (Fig. 4g). These findings demonstrate that at 21 days post-amputation, the difference in growth rates between proximal and distal blastemas is not due to differences in the rate of cell division, but is instead due to the percentage of cells in the blastemas that are actively dividing (twice as many in the proximal than in the distal blastema).

### Calculations of the founding cell population for the tail blastema and two-factor model for tail blastema growth

With cell cycle parameters in hand, we sought to determine the total number of progenitor cells that are recruited from the mature tissue to participate in tail regeneration from proximal and distal amputations. To accomplish this, we counted the total number of cells present at day 10 in regenerated tails from proximal and distal amputations. Based on the cell cycle lengths and growth fractions determined in Fig. 4g,h at day 10, we calculated the number of progenitor cells that would be required to produce the observed number of cells in the regenerated tails (for the mathematical model see Methods).

In blastemas arising from proximal amputations, we found an average of 39,982 ± 5024 cells in the regenerated tissue (Table 1) and calculated that 12,552 ± 1577 cells contributed to the regenerated tissue. When we counted the number of mesenchymal cells at the amputation plane, we found 794 ± 88 cells in a cross sectional area of a proximal amputation. Based on this, we extrapolate (see Methods) that cells coming from approximately 348 ± 82 μm behind the amputation plane would contribute to the blastema, if one assumes that all cells at the amputation plane contribute to the regenerate and divide. In fact, the percentage of cells that contribute is likely to be lower, as cell death likely occurs at the site of amputation and not all cells may become proliferative to the same extent. Therefore, we conclude that the zone of activation extends *at least* 350 μm back from the amputation plane. This zone length is consistent with a previous report demonstrating that after tail amputation, spinal cord progenitors in a 500 μm zone proximal to the amputation plane are responsible for reconstituting the entire regenerated spinal cord [7].

**Table 1:** Calculation of the progenitor cell number and spatial extent

	Cell number in day 10 regenerate	Progenitor cell number	Cells at amputation plane	Distance from amputation plane
Proximal	39,982 ± 5024	12,552 ± 1577	794 ± 88	348 μm ± 82 μm
Distal	22,154 ± 2862	7485 ± 967	376 ± 65	438 μm ± 132 μm

Regenerated tails from proximal and distal blastemas were collected on day 10 post-amputation, fixed, cryosectioned and total mesenchymal cell numbers counted (see Methods). The table compares the total cell counts, the calculated number of progenitor cells required to give rise to the total cell number within 10 days (accounting for all proliferation parameters, see Methods), the counted cell numbers within the amputation plane and the calculated spatial extent of the progenitor pools for both amputation levels. Despite several individual parameters differing between amputation levels, the distances the progenitor pools extend proximal to the amputation plane are similar within the confidence intervals

In blastemas arising from distal amputations, we found an average of 22,154 ± 2862 cells in the regenerated tissue (Table 1). Based on calculations as described above, this would represent a founding progenitor cell pool of 7485 ± 967 cells. Interestingly, the cell count in the cross-sectional area of the distal amputation is 376 ± 65 compared to 794 ± 88 for the proximal amputation plane. This different cross-sectional area and the different progenitor cell number lead to an extrapolation that cells come from 438 ± 132 μm behind the amputation plane, similar within the confidence interval to the figure found for the proximal amputation. Taken together, these calculations suggest that the larger proximal blastema at day 15 arises from a larger initial founding population due to a larger cross sectional area at the amputation plane compared to a distal amputation. Most importantly, amputation-induced signals appear to activate a 400 μm long progenitor zone, likely independent of the cut position and similar for muscle and spinal cord regeneration [7].

**Fig. 5:**
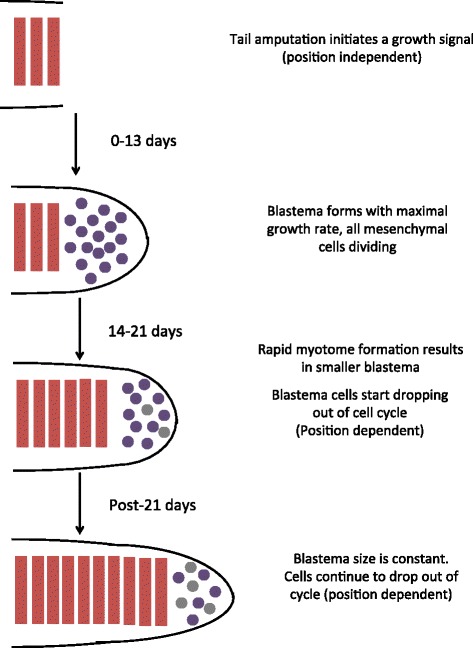
Schematic of growth phases (rows), growth characteristics and position-dependent growth regulation for tail regeneration

From these calculations, we propose a two-stage model for blastema cell proliferation and myotome formation (Fig. 5). After amputation, early injury responses cause production of a signal that induces local recruitment and rapid proliferation of blastema cells for approximately 2 weeks. This signal is likely independent of position of the amputation along the length of the tail. Based on our calculations, cells are recruited from a zone that extends a similar distance back from the amputation in proximal and distal cuts. Since the proximal amputation site has a thicker cross-sectional area than the distal site, the proximal blastema is established with a larger number of founding cells. Proliferation of all these founding cells for 2 weeks results in a bigger proximal than distal blastema, given their cone shape. In our view, the larger proximal blastema that forms up to day 15 is a fortuitous outcome related to tail morphology and is not essential for the proximal blastema to ultimately regenerate more tail tissue. After day 15, a proximal-to-distal myotome differentiation wave initiates, which is faster in the proximal versus distal cut site until a steady state blastema size is reached by day 21 (Fig. 2b,c). Concomitantly, the blastema cells appear to start exiting the cell cycle faster in the distal versus the proximal blastemas, so that by day 21, a higher proportion of proximal blastema cells remain proliferative (Fig. 4h). We suggest that between day 15 and 21, the initial injury-induced growth inducing signal wanes, revealing a second growth-sustaining signal whose strength is position-dependent along the anterior-to –posterior axis. It is the second, position-dependent growth phase that ultimately dictates the final outcome of regenerate length.

## Discussion

Here we have measured the kinetics of tail regeneration, comparing two levels of amputation. We focused our measurements on the growth of the blastema, and on the differentiation of myotomes in order to compare regeneration at different amputation levels. We further coupled this analysis of overall tail regeneration with changes in cell cycle kinetics over time using the cumulative BrdU methodology. To our knowledge it is the first time that measurements integrating blastema size, myotome differentiation and cell cycle parameters have been performed. From these measurements, we conclude that proximal and distal blastemas both undergo “maximum” growth where all cells are dividing for almost 2 weeks before onset of myotome organization, which occurs in an anterior to posterior wave and consumes most of the blastema except an undifferentiated terminal tip that reaches a common size by day 21. These observations suggest that from day 15 to day 21 there is a counter-action of pro-differentiation signals from the amputation plane versus anti-differentiation signals at the end of the blastema (possibly but not necessarily emanating from the wound epidermis). By day 21, a “steady state” balance is reached that allows maintenance of an undifferentiated blastema at the tail tip. However, the cells in a proximal blastema are withdrawing from the cell cycle at a slower rate than distal blastemas. This is presumably an intrinsic, level-specific property of the blastema cells. It is not known if the level-specific property is production of a limiting amount of an extrinsic growth factor, or an inherent difference in the proliferative potential of the cells. Heterotopic and heterochronic transplantations may provide insight into this issue in future.

Our observation that the time of myotome differentiation onset is similar between proximal and distal cuts is consistent with previous descriptions of tail and limb regeneration indicating that proximal and distal blastemas undergo the major stages of regeneration at similar time points. Other studies measuring elongation rates of proximal versus distal regenerating tail tissue in *Ambystoma punctatum* and *Rana Clamitans* noted level-specific differences in elongation rates and completion times [8, 9]. Our data would be consistent with level-specific differences in elongation rate, but not in completion time. However the tail regeneration observed in *Rana Clamitans* tadpoles was never truly complete, which perhaps accounts for the observed differences in “completion” time. In terms of cell cycle kinetics our results are relatively consistent with previous observations. Previous tritiated thymidine experiments concluded that the cell cycle length in axolotl limb blastema cells is approximately 53 h, slightly shorter than our current measurements [1]. In this study, the cell cycle length was measured by examining the percentage of mitoses that harbored tritiated thymidine label at different time points after thymidine induction. This difference in method may account for the observed differences from our study, or there may be differences between limb and tail blastema cells. Tomlinson and Barger performed cumulative thymidine incorporation studies in axolotl limb blastema cells and obtained similar growth curves but did not calculate cell cycle length. Rather they interpreted their data to indicate a “punctuated cell cycle” in which the S-phase cells identified by a single pulse of thymidine reflect rapidly dividing cells. Those cells that did not incorporate thymidine in single pulse time points were deemed to be “paused”, without a calculation of the pause time.

It is interesting to contemplate the commonalities and differences between establishing segment number during development versus during regeneration. Clearly there are structural differences. For example, myotome differentiation during tail regeneration occurs in the absence of a somite intermediate. An interesting question is whether regeneration is comparable to the myotome organization observed in zebrafish lacking Notch signalling, where no somites form, but adaxial cells template the formation of myotomes [10]. Nonetheless, segmentation during embryogenesis and regeneration share a common feature in which a proliferating, undifferentiated field of mesenchyme undergoes proliferation with an anterior wave of differentiation. During development, retinoic acid appears to be a pro-differentiative signal whereas WNT and FGF signals act as anti-differentiative signals [11, 12]. Interestingly, inhibition of WNT or FGF signalling stops tail regeneration [13]. In future work it will be important to determine whether such factors are involved in the level specific differences in cell cycling within the undifferentiated mesenchyme, an issue that is also not yet resolved during primary axis formation.

## Conclusions

The quantitative comparison of blastema size, cell cycle kinetics and myotome differentiation rate for 4th and 16th post-cloaca level tail blastemas presented here suggest that there are two separable phases of blastema growth. The first is level-independent, with cells displaying unrestrained proliferation. In the second phase, the level-specific growth is revealed, where larger fractions of cells remain in the cell cycle for proximal regenerates as compared to distal regenerates at the same time.

## Methods

No consent statements were required for the work.

### Care of axolotls and ethics approval

All experiments utilized white mutant (d/d) animals that were bred in the laboratory. Animals were kept in tap water in a climate-controlled room at 20 °C. Animals were kept in separate containers for the duration of all experiments. Prior to amputation, tissue collection, or microscopic analysis, animals were anesthetized in 0.01 % benzocaine. The work was performed under an approved license number 24–9168.11-9/2009-3 from the Animal Commission of the Landesdirektion Sachsen.

Proximal tail blastemas were defined as blastemas arising from amputation of the tail at the level of the 4th myotome caudal to the cloaca (see Fig. 2). Distal tail blastemas were defined as blastemas arising from amputation of the tail at the level of the 16th myotome caudal to the cloaca. In both cases, all tissue caudal to the last myotome in the amputated tail was considered part of the blastema.

### Myotome scoring

Myotomes were counted in live, anesthetized animals using an Olympus Stereo SZX12 dissecting microscope, which provides oblique illumination from below the specimen, allowing visual detection of newly formed myotomes in the tail. Regenerated tail myotomes are often irregularly spaced and can partially overlap; in these situations myotome number was assessed by scoring the number of myotomes directly adjacent to the cartilage rod ventral to the spinal cord.

### Myotome formation rate

For each individual animal, we calculated the myotome formation rate in a given time-interval by linear regression of the myotome counts vs time. For the different experimental conditions we then report the mean myotome formation rate of the corresponding replicates and estimate the 68 % confidence interval with the standard error of the mean.

### Cumulative BrdU incorporation

Cumulative BrdU labeling was performed as described in [6] with the following modifications. Approximately 30 μL of BrdU (8 mM in APBS containing Fast Green) was injected peritoneally in anesthetized axolotls 2 to 4 cm in length. Animals were wrapped in a moist paper towel for approximately 5 min to allow the wound to heal, then returned to tapwater. At each timepoint, all animals were injected with BrdU, and 1 h later tails were collected from a subset of the animals. Tail tissue was fixed overnight at 4 °C in MEM buffer containing 3.7 % formaldehyde. Fixed tissue was then washed with PBS and embedded in OCT, 7.5 % gelatin, or paraffin prior to sectioning by standard methods [7].

### BrdU detection

BrdU-positive nuclei were detected by standard immunofluorescence techniques [7]. Tail sections (10 μM) were washed three times in PBS, incubated in 2 N HCl at 37 °C for 15 min, then washed three times in PBS to remove the HCl. Samples were then blocked for 1 h at room temperature with blocking buffer (PBS, 0.03 % Triton X-100, 10 % goat serum). Samples were then incubated overnight at 4 °C in blocking buffer containing a rhodamine-conjugated mouse monoclonal antibody against BrdU clone Bu20. Samples were then washed 3× with wash buffer (PBS, 0.03 % Triton X-100), and nuclei were stained by incubation in PBS containing Hoechst (1 μg/ml) for 20 min at room temperature. Samples were washed with PBS, sealed and viewed using a Zeiss Axiovert microscope. To enable the comparison of proliferation characteristics between different time points and conditions and to ease data analysis by fixing the total cell number, a single section at the same spatial level, marked by the tip of the cartilage rod, was analyzed per animal.

### BrdU data analysis

As a robust framework to probe cell cycle parameters, we chose a simple model with only two cell populations (cycling and quiescent) that is defined by just 3 parameters (cell cycle duration *T*_*C*_, S-phase duration *T*_*S*_ and growth fraction *GF* of cycling cells among the fixed total number of cells) [6]. This model assumes 1) that all cycling cells within the analyzed section occur homogenously distributed over the cell cycle phases and have identical cell cycle parameters, 2) a fixed total cell number and 3) a constant fraction of quiescent cells. Then, the BrdU labeling index is predicted to increase linearly with time until saturation at the value *GF*. We fitted the model using the method of least squares utilizing the function *scipy.optimize.brute* (http://www.scipy.org/). We determined parameter estimates (mean of the bootstrap samples) and 68 % confidence intervals (mean ± standard deviation of the bootstrap samples) by bootstrapping with a case resampling scheme of 10,000 resampled datasets. To decide whether parameters in two experimental conditions are significantly different, we calculated the difference between the parameters’ estimates (*d*) and estimated its 68 % confidence intervals (*Δd*) by linear error propagation. Then, two the parameters are significantly different with *p* < 0.05 if |*d*| - 2*Δd* > 0 and with *p* < 0.001 if |*d*| - 3*Δd* > 0.

### Total cell count protocol

Tail tissue was collected after 10 days of regeneration from animals amputated at either the 4th or the 16th myotome caudal to the cloaca. The tissue was fixed overnight at 4 °C in MEM buffer containing 3.7 % formaldehyde, washed 3× in PBS, then incubated in 10 % sucrose, 20 % sucrose, 20 % sucrose containing 3.5 % gelatin, and finally 20 % sucrose containing 7.5 % gelatin. The tissue was then embedded in gelatin and 10 μm cryosections were collected along the entire regenerate. Sections were stained with Hoechst as described above, and every 8th section was scored for the total number of nuclei present in the section (excluding epidermis, spinal cord, and cartilage rod nuclei). The total number of mesenchymal cells within all consecutive sections was obtained by multiplying the average nucleus count per section by the total number of sections that were collected for each tail. Because the average nucleus in the blastema is approximately 22 μm in diameter and each section was 10 μm thick, the total number was divided by 2.2 to account for parts of each nucleus that extend over 2 or 3 consecutive sections and generated a 22/10 higher apparent average nucleus count per section.

### Mathematical model for progenitor pool and length estimation

The considered cell numbers are large enough (>1000) to warrant an averaged and deterministic description. Given the total cell count *N(t)* in the regenerate (not including any cells anterior to the amputation plane), growth fraction *GF* and cell cycle length *T*_*C*_ at *t* = 10d, the progenitor pool size *N*_*0*_, located anterior to the amputation plane at *t* = 0d (when *N(t* = 0d*)* = 0) and including a fixed number of quiescent cells *N*_*q*_ = (1-*GF*)*(*N(t* = 10d*)* + *N*_*0*_), can be calculated as follows:

$$ N(t)+{N}_0={N}_q+\left({N}_0-{N}_q\right)\cdot {2}^{\left(t/{T}_C\right)} $$

which yields the progenitor pool size

$$ {N}_0=N(t)\cdot \left(1+\left(1/\mathrm{G}\mathrm{F}-1\right)\cdot {2}^{\left(t/{T}_C\right)}\right)/\left({2}^{\left(t/{T}_C\right)}-1\right). $$

The resulting parameter dependencies in this formula can be understood intuitively as follows. The sum in the numerator accounts for the mix of cycling and quiescent cells and is larger than 1 corresponding to a larger progenitor pool in case of more quiescent cells. This sum simplifies to 1 if the growth fraction GF is 100 %. The subtraction of 1 in the denominator accounts for the cell number that replaces the initial progenitor pool at its location anterior to the amputation plane.

The 68 % confidence interval was calculated from Δ*N*_*0*_ = *N*_*0*_ * (Δ*N(t)*/*N(t))* assuming the animal-to-animal variability in *N(t)* to be the dominating source of uncertainty.

The length of the tissue anterior to the amputation plane which harbors the inferred progenitor pool can be calculated by dividing *N*_*0*_ by the nucleus density per length. The nucleus density per length differs between proximal and distal cut locations, mostly as a consequence of 3d tail geometry. We counted the number of nuclei *N*_*a*_ in single 10 μm thick sections at the amputation plane of each animal and divided the average (reported in Table 1) by the correction factor of 2.2 accounting for the same nuclei occupying multiple sections, see above. The progenitor zone length *D* is then obtained from

$$ D={N}_0/\ \left(\left({N}_a/ 2.2\right)/10\ \upmu \mathrm{m}\right). $$

Error estimates were propagated by adding the relative errors of input variables, hence Δ*D* = *D* * (Δ*N*_*0*_/ *N*_*0*_ + Δ*N*_*a*_/*N*_*a*_), and an additional error may result from the estimate of cell density per length.
